# Towards an Evolutionary Model of Transcription Networks

**DOI:** 10.1371/journal.pcbi.1002064

**Published:** 2011-06-09

**Authors:** Dan Xie, Chieh-Chun Chen, Xin He, Xiaoyi Cao, Sheng Zhong

**Affiliations:** 1 Department of Bioengineering, University of Illinois at Urbana-Champaign, Urbana, Illinois, United States of America; 2 Department of Biochemistry and Biophysics, University of California, San Francisco, California, United States of America; 3 Center for Biophysics and Computational Biology, University of Illinois at Urbana-Champaign, Urbana, Illinois, United States of America; Weizmann Institute of Science, Israel

## Abstract

DNA evolution models made invaluable contributions to comparative genomics, although it seemed formidable to include non-genomic features into these models. In order to build an evolutionary model of transcription networks (TNs), we had to forfeit the substitution model used in DNA evolution and to start from modeling the evolution of the regulatory relationships. We present a quantitative evolutionary model of TNs, subjecting the phylogenetic distance and the evolutionary changes of *cis-*regulatory sequence, gene expression and network structure to one probabilistic framework. Using the genome sequences and gene expression data from multiple species, this model can predict regulatory relationships between a transcription factor (TF) and its target genes in all species, and thus identify TN re-wiring events. Applying this model to analyze the pre-implantation development of three mammalian species, we identified the conserved and re-wired components of the TNs downstream to a set of TFs including Oct4, Gata3/4/6, cMyc and nMyc. Evolutionary events on the DNA sequence that led to turnover of TF binding sites were identified, including a birth of an Oct4 binding site by a 2nt deletion. In contrast to recent reports of large interspecies differences of TF binding sites and gene expression patterns, the interspecies difference in TF-target relationship is much smaller. The data showed increasing conservation levels from genomic sequences to TF-DNA interaction, gene expression, TN, and finally to morphology, suggesting that evolutionary changes are larger at molecular levels and smaller at functional levels. The data also showed that evolutionarily older TFs are more likely to have conserved target genes, whereas younger TFs tend to have larger re-wiring rates.

## Author Summary

DNA evolution models made invaluable contributions to comparative genomic studies. Still lacking is an evolutionary model of transcription networks (TNs). To develop such a model, we had to forfeit the substitution model used in DNA evolution and to start from modeling the evolution of the regulatory relationships, and then subject the phylogenetic distance and the multi-species DNA sequence and gene expression data to one probabilistic framework. This model enabled us to infer the evolutionary changes of transcriptional regulatory relationships. Applying this model to analyze three yeast species, we found the anaerobic phenotype in two species was associated with the evolutionary loss of a larger *cis-*regulatory motif than previously thought. Analyzing three mammalian species, we found increasing conservation levels from genomic sequences to transcription factor-DNA interaction, gene expression, TN, and finally to morphology, suggesting that evolutionary changes are larger at molecular levels and smaller at functional levels. We also found that evolutionarily younger TFs are more likely to regulate different target genes in different species.

## Introduction

Biologists have long sought to dissect what genes and what changes to their coding and regulatory sequences are responsible for the diversity of life. It has been argued that morphological traits evolve to a large extent through changes in transcription networks (TNs) that regulate gene expression patterns [1], [2]. However, except on a relatively small set of well characterized enhancers, it appears difficult to quantitatively analyze cis contribution to TN evolution (see reviews [2], [3], [4]). This is in part due to the computational difficulties in finding *cis-*regulatory sequences or enhancers [5], assessing the binding affinity of an enhancer to a set of transcription factors (TFs) [6], and associating enhancer binding affinities with gene expression levels [7]. Still lacking are principled approaches and evolutionary models to quantitatively analyze the effects of changes in *cis-*regulatory sequences, gene expression, and TNs (Table S1).

The question that inspired us to model TN evolution is the conservation of early embryonic development in mammals. The earliest stages of embryonic development are thought to be highly conserved among placental mammals, because these species all progress through the same morphologic stages before implantation. This traditional view is challenged by recent reports on large inter-species differences in gene expression [8] and TF binding patterns [9]. During pre-implantation development (PED), an unexpected fraction of 40% of orthologous gene triplets exhibited different expression patterns among humans, mice and cattle [8], accompanied by an even more unexpected fraction of 95% of the binding sites of the core TFs, Oct4 and Nanog, in human and mouse embryonic stem (ES) cells not being located in orthologous genomic regions [9]. Large interspecies differences of gene expression [10] (Figure S1) and transcription factor binding sites [11] were also observed in matched tissues in vertebrates. How can we understand the discrepancy between morphological conservation and molecular differences across species? We hypothesized that the structure of TN, *i.e.* the regulatory relationship between transcription factors (TFs) and target genes, may be more conserved than suggested by TF binding site (TFBS) or gene expression data. For instance, some TFBSs turn over quickly during vertebrate evolution, without necessarily changing TF-target relationships. To test this hypothesis and to provide a general tool for studying TN evolution, we set off to develop an evolutionary model for TN structure based on multi-species genome sequence and gene expression data.

Previous work has made excellent progress in modeling the evolution of regulatory genomic sequences. Earlier attempts were focused on identifying putative regulatory sequences highly conserved across multiple species [12], [13] or containing conserved TF binding motifs [14], [15]. Recent efforts extended the earlier work by accommodating lineage-specific changes and alignment errors [16], [17], incorporating the modular structure of regulatory sequences [5], [18], and direct modeling of TF occupancy [6], [19]. Although these models have been evolved to accommodate many evolutionary events on DNA, it is difficult to extend these models to incorporate the evolutionary changes of non-genomic features. This difficulty can be partially appreciated by noticing that even in a single species, the state-of-the-art models resort to a regression strategy to incorporate DNA sequence and gene expression into one model (see [20] and references within).

In parallel to sequence evolution models, evolutionary models for gene expression are being developed and tested. A neutral evolutionary model for gene expression was proposed [21], enabling statistical tests for evolutionarily selected genes [22]. A major challenge in comparing expression data between organisms is that gene expression is not static and the level of expression is influenced by external conditions. A prominent approach to circumvent this difficulty is to compare co-expression patterns rather than the expression of individual genes [23], [24]. This approach has recently been formalized into evolutionary models of co-expression networks, which are based on explicitly stated probabilistic rules [25], [26]. These evolutionary models did not explicitly study the evolution of genomic regulatory relations. In fact, while expression data can be useful in predicting co-regulation (in particular, among target genes of the same TF), such data alone can hardly predict which gene is regulated by which TF as the correlation between expression patterns of a TF and that of its targets may not be pronounced [27], [28].

**Figure 1:**
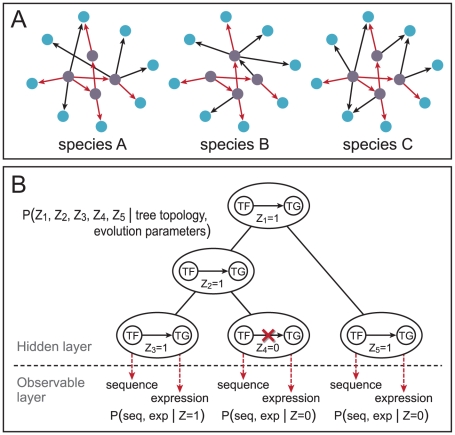
A model for TN evolution. (A) An example of evolving TNs. Transcription factors and target genes are depicted in purple and blue nodes. Conserved and species-specific regulatory relationships are depicted with red and black arrows. (B) The regulatory states are modeled as a continuous time Markov chain, and the regulatory sequence and the gene expression data are emitted from the hidden regulatory states.

In order to build an evolutionary model of TNs, we had to forfeit the substitution model used in DNA evolution and the co-expression model used in expression evolution, and to start from modeling the evolution of the regulatory relationships. In this paper we present a quantitative evolutionary model of TNs, subjecting the changes of *cis-*regulatory sequences, gene expression and network structure to one probabilistic framework. This model aims to address the following question: with the genome sequences and gene expression data from multiple species, can the TF-target gene relationships be derived in all these species? Taking advantage of the multi-species data and based on the maximum-likelihood principle, this model predicts the evolutionary changes of TF-target relationships, *i.e.* re-wiring of TNs (Figure 1). The major benefits of this model include: 1) it takes advantage of the possible synergism between genome sequence and gene expression data. For instance, if a gene is predicted to be a TF target from sequence data, other genes co-regulated with this gene, according to the expression data, may also be a target. 2) By analyzing data in the evolutionary context, the model is still able to utilize pattern of conservation to predict regulatory relationship (similar to the evolutionary models of regulatory sequences we discussed earlier), while allowing for lineage-specific changes. 3) The unified probabilistic framework allows us to quantify the extent of changes and the uncertainty of the inference results.

## Results

### Simulation study

We simulated a series of six synthetic datasets and used 5-fold cross validation to test the model (see Text S1). Cross-validation results showed almost identical prediction accuracies in training and testing datasets, across all simulated noise levels in sequence and in expression data, and across all choices of the weight parameter (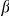), suggesting that it is fairly difficult to make model overfit (Figure S2).

### Transcriptional re-wiring in yeasts – a test of the model

To test the TN evolution model with real data, we used the recent discovery of a re-wiring event in yeast species. We wanted to use the analysis of this relatively well described re-wiring event to test the validity and precision of the new model.

Genes coding for mitochondrial and cytoplasmic ribosomal proteins display a strongly correlated expression pattern in *Candida albicans*, but this correlation is lost in the fermentative yeast *Saccharomyces cerevisiae*. Ihmels *et al.* associated this change in gene expression with the loss of a specific *cis-*regulatory element, AATTTT, from dozens of mitochondrial ribosomal protein (MRP) genes [29]. We attempted to reproduce this finding and potentially explore it in greater details with new data and the TN evolution model. Because the inferred loss of the *cis-*regulatory element happened after the separation of aerobic and anaerobic yeast species, we chose to analyze two anaerobic species, *Saccharomyces cerevisiae*, and *Candida glabrata*, and one aerobic species *Candida albicans*. We identified in the three species the orthologs of 51 MRP genes, 58 rRNA genes, and 73 stress response (STR) genes. While the three gene sets formed their individual expression clusters in *S. cerevisiae* and *C. glabrata*, MRP and rRNA genes appeared to be co-expressed in *C. albicans* (Figure S3-A). An enriched sequence motif was found by MEME [30] in the promoters of rRNA genes of all three species, as well as in the promoters of the MRP genes in *C. albicans* (Figure S3-B New Motif). We hypothesized that this motif may represent the binding specificity of a conserved TF, which we termed TF_a_. If Ihmels *et al.*'s finding can be reproduced, there should exist a TF that regulates MRP genes only in *C. albicans* but not in anaerobic species. We let our evolutionary model predict the transcriptional targets of TF_a_ in all three species. We compared the model-predicted regulatory relationships to Ihmels' theory and found strong consistency (Figure S3-B) (Methods). This suggests that the TN evolution model captured the re-wiring of MRP genes as Ihmels *et al.* reported and provides additional support to the hypothesis that the re-wiring event is correlated with the divergence of aerobic and anaerobic species.

Since the TN evolution model enabled the analysis to include the expression and the sequence data of a third species, *C. glabrata*, which was not present in Ihmels' analysis, we expected the model-based analysis to reveal more details regarding TF_a_ and its regulatory rules. To this end, we asked whether the 6bp *cis-*regulatory element, AATTTT, identified by Ihmels *et al.* was optimal. We compared AAATTTTT (new) and AATTTT (Ihmels) by the number of target genes they can correctly predict in the three species. The new motif was more informative in predicting both the target genes and the non-target genes (Figure S3-B, panel ALL, Table S2). To assess the robustness of this result, we investigated every gene group (MRP, rRNA, STR), and we varied the weight of expression data (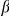) used in the model. The new motif better distinguished the target and the non-target genes in all the settings tested (Figure S3-B), suggesting the new motif is a more faithful representation of TF_a_'s binding preference. These results corroborated our expectation that the precision of the model is suitable for making discoveries.

### How different are the TNs among mammals during early embryonic development?

To investigate the discrepancy between morphological conservation and molecular differences in mammals, we hypothesized that the TN structure does not evolve as quickly as the TF binding sites and gene expression. In other words, we hypothesized that although there are substantial amounts of TFBS turnovers and gene expression changes across mammals, there are fewer changes in TN structure, *i.e.* TF-target regulatory relationships.

**Figure 2:**
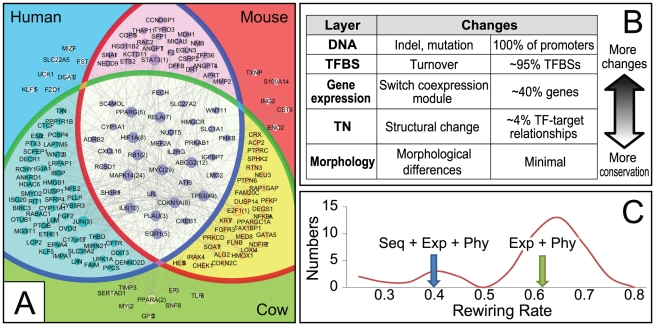
Conservation and rewiring of Oct4 downstream TN. (A) Venn diagram of model-inferred Oct4 target genes in three species. Each node is a gene and each edge represents a transcriptional regulatory link. When a set of genes are linked to a regulatory hub gene, this set of genes is suppressed into one node, annotated with the name of the hub gene followed by the number of other genes transcriptionally linked to the hub. (B) Inferred conservation levels for DNA, TFBS, gene expression, TN, and morphology. (C) Comparison of model-inferred re-wiring rates with (Exp+Seq+Phy) and without (Exp+Phy) DNA sequence data. The re-wiring rate of TF was calculated as the percentage of non-conserved TF-target relationships among all TF-target relationships. A background distribution (red curve) of the re-wiring rate is derived from randomly permuting the rows and columns of the Oct4 position specific score matrix (PSSM) and feeding the permutated PSSM to the model with unperturbed sequence and gene expression data.

To test this hypothesis, we applied the TN evolution model to analyze the sequence and expression data in PED of humans, mice and cattle. Out of a total of 7046 orthologous gene triplets in the three species, 1489 of them fell into some co-expression modules (had non-constant expression and were not clustered as singletons). We chose Oct4 as the TF of focus in this study. The output of our evolutionary model is the regulatory relationship between a TF, Oct4 in this case, and every gene in every input species. Among the 1489 orthologous gene triplets, 823 (55.3%) were predicted to be regulated by Oct4 in all three species, and 113 (7.6%) were predicted to be only regulated in one species [nodes, Figure 2A]. In particular, only 40 (2.7%) orthologous triplets were regulated by Oct4 specifically in humans. This estimated fraction (2.7%) is much smaller than the fraction of genes with human-specific PED expression (45%, p-value<1E-10) [8], which in turn is much smaller than the fraction of human-specific Oct4 binding sites (95%, p-value<1E-10) [9] [Figure 2B].

To assess whether the model-inferred *smaller* interspecies difference in TF-target relationship than the previously reported interspecies difference of gene expression is due to model priori, we did two control experiments. First, the gene expression data alone was fed to the same evolutionary model, which led to an estimated 62.3% re-wiring rate [Figure 2C, Exp+Phy]. In this case the estimated re-wiring rate should be interpreted as the percentage of genes with unconserved expression patterns among the three species, which is consistent with previously reported 55% [8]. Second, both sequence and expression data were fed to the evolutionary model together with randomly permuted Oct4 DNA binding motifs, which led to a distribution of the estimated re-wiring rates [Figure 2C, red curve]. The mode of this distribution was 0.66, and 82.2% of the estimated rates lay between 0.5 and 0.8. Both control experiments subjected gene expression data to the same model priori as the TF-target analysis, and consistently reported larger estimated interspecies changes of gene expression than TF-target relationship, considering the model inferred 44.7% (100%-55.3% conserved targets) re-wiring rate for Oct4.

We then asked to what extent the interspecies difference in Oct4 target genes affects the downstream gene regulatory networks. We mapped protein-protein interactions and transcriptional regulatory relationships among all the genes [Figure S8, Figure 2A]. Among all the possible protein-protein interactions (PPIs) [31], [32], [33], [34], 134 interactions were found between the genes that were predicted to be conserved targets in all three species, whereas 0 interactions were found between the genes that were specifically regulated by Oct4 in any one species [gray edges, Figure S8] (Chi-square test p-value = 6.6E-210). Among all the transcriptional regulatory relationships [35], [36], 270 regulatory relationships were found between the genes that were predicted to be conserved targets in all three species, whereas 1 interaction was found between the gene that were specifically regulated by Oct4 in only one species [gray edges, Figure 2A] (Chi-square test p-value = 8.7E-307). In summary, the data above suggest that compared to the changes in TFBS and gene expression, the transcriptional targets of Oct4 are more conserved, and so are the interaction and regulatory relationships of these Oct4 target genes [Figure 2B, Figure S6].

### Evidence for re-wiring of TNs in PED

**Figure 3:**
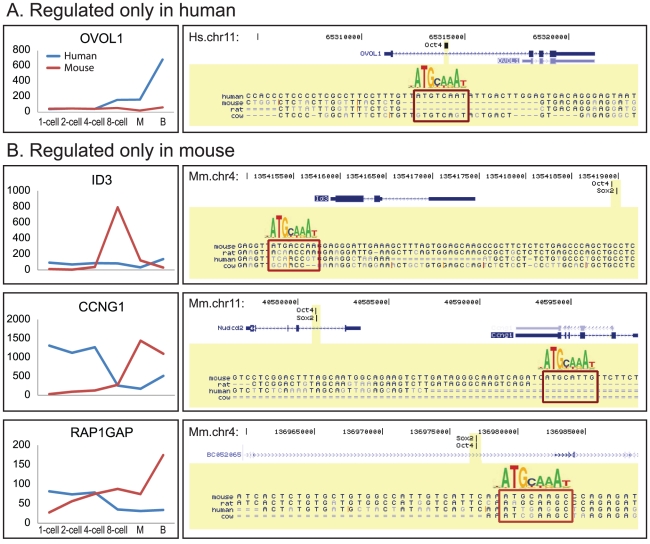
Examples of model-inferred species-specific target genes of Oct4 in humans (A) and mice (B). Oct4 is zygotically expressed (4–8 cells), and it is strongly increased at the late stages of pre-implantation development, including morula (M) and blastocyst (B). The target genes' expression is zygotically activated in a species-specific manner (Left panel). The species-specific binding of Oct4 to target genes is observed in ChIP-seq experiments (TFBS track, right panel). The genomic sequences of the Oct4 binding regions are shown in yellow, with Oct4 binding motifs shown in red boxes.

There is as yet no proven example of TN re-wiring events reported for early development in mammals, like the example Ihmels *et al.* demonstrated in yeast species. We wanted to identify a few concrete cases of TN re-wiring events. The TN evolution model provides a systematic approach to look for TN re-wiring events (Text S2 and Figure S4). Our model predicted 40 genes as Oct4 targets in humans but not in mice, and vice versa for 24 genes [Figure S5]. We applied two further criteria to these 64 genes to select for re-wiring events with the strongest evidence. First, because *Oct4* itself shows an upward trend of expression during PED, peaking at the blastocyst stage in humans and at the morula stage in mice [8], we selected the genes with clear up-regulation in the late stages of PED. Second, we selected the genes whose predicted Oct4 binding regions harbored clear gain or loss of Oct4 binding motifs. These selections produced four TN re-wiring events associated with the human-specific regulation of *OVOL1* by Oct4 and mouse-specific regulation of *Id3*, *Ccng1*, and *Rap1gap* [Figure 3]. Because embryonic stem (ES) cells were derived from the inner cell mass of blastocyst stage embryos (the last developmental stage of PED), we speculated that TN re-wiring events could be corroborated by gain or loss of Oct4 binding in ES cells. ChIP-seq data in human and mouse ES cells were consistent with this hypothesis [37], [38] [“TFBS” track, Figure 3]. By reconstructing the ancestral sequence, we identified the evolutionary events including indels and mutations that mediated the TFBS turnovers [Figure S5]. In particular, the birth of the Oct4 TFBS near *Id3* gene appears to be mediated by a 2bp deletion from ACAgtACCGTG (ancestral) into ACAACCGTG (murine) [Figure S5-B]. TFBS birth by deletion has rarely been reported in vertebrates.

### Quantitative clues about cis changes that affect TN re-wiring

**Figure 4:**
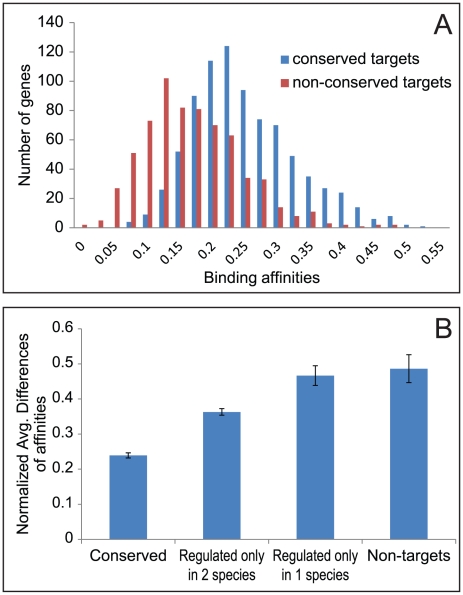
Relationship between conservation and binding affinities. (A) The distribution of the average binding affinities between Oct4 and orthologous regulatory sequences in three species. (B) Interspecies difference in binding affinities of the 20 kb upstream sequences of orthologous genes. The orthologous genes are put into four categories of conservation. Error bars show 95% confidence intervals.

A long standing question is to what extent the evolutionary changes of the TF-target relationship are associated with cis changes. In other words, for a conserved TF, can we use the cis changes to infer changes in regulatory relationships? Except for testing done on a small set of experimentally characterized enhancers [39], genome-wide analysis attempts seemed to provide negative answers. For example, changes in DNA binding motifs do not seem to correlate well with changes in TF-DNA binding [40], and loss (gain) of in vivo TFBS may not affect target gene expression because they can be compensated by gain (loss) of in vivo TFBS in other regulatory regions of the same target gene [9]. The TN evolution model enabled us to revisit this question. In the case of Oct4 regulated genes, the conserved target genes (regulated by Oct4 in three species) harbored *cis-*regulatory regions (20 k bp flanking TSS) with larger binding affinities to Oct4, as compared to the non-conserved target genes [Figure 4A]. This is consistent with the fact that motif information was used in the model. Moreover, the interspecies difference in the binding affinities, as determined by the Oct4 motif and the *cis-*regulatory regions, is inversely correlated with the conservation level of the TF-target relationship [Figure 4B]. More specifically, the average interspecies cis difference in the target genes that are conserved in all three species is 55% of the average interspecies cis difference of the target genes conserved in two species (not a target gene in the third species) (p-value = 5.15407E-76). The latter difference is in turn 75% of the average interspecies cis difference of the target gene in only one species (not a target in the other two species) (p-value = 2.877E-17); However, it is not statistically different from the interspecies cis difference of non Oct4 target genes (p-value = 0.1204).

### Do younger TFs have larger re-wiring rates?

**Figure 5:**
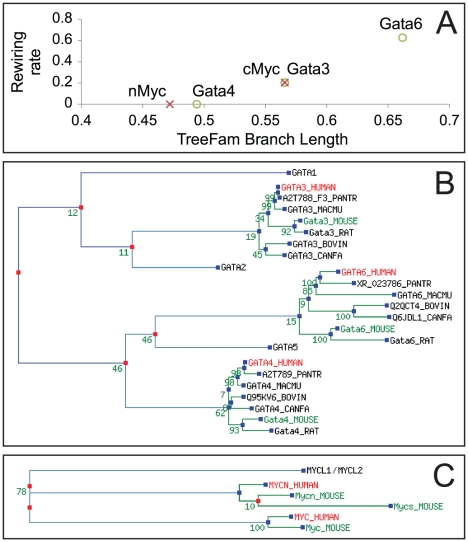
Re-wiring rate against divergence time. (A) Re-wiring rate against phylogenetic distance. Circles: Gata3/Gata4/Gata6 genes. Cross: cMyc(MYC)/nMyc(MYCN) genes. TreeFam branch length of a gene is the distance between the speciation event of the gene and the first duplication event in the paralogous family. (B, C) Phylogenetic trees of the Gata and the Myc families in mammals. Blue dot: gene speciation event. Red dot: gene duplication event. Branch lengths are estimated for the consensus tree of bootstrapped trees using HKY model. Numbers on branching events are the support numbers to the consensus tree in 100 bootstrapped trees.

Some TFs are evolutionarily younger than others. We asked whether the divergence time of a TF from its ancestor is correlated with its re-wiring rate. The re-wiring rate of a TF is defined as the percentage of non-conserved TF-target links among all TF-target links of a TF. Since our model infers the target genes of a TF in every species fed to the model, re-wiring rate can be directly derived from the model output. An extensive survey of TFs that may regulate cell fate decisions in mouse PED reported 29 TFs [41], among which there were two sets of paralogous genes including Gata3/Gata4/Gata6 and cMyc/nMyc [Figure 5]. Using the HKY DNA substitution model [42] implemented in TreeFam [43], we inferred the divergence time of each TF to the closest common ancestor of the paralogous group [Figure 5B,C]. In all four comparisons (cMyc vs. nMyc, Gata4 vs. Gata3, Gata6 vs. Gata3, Gata6 vs. Gata4), the TF with a shorter divergence time always showed a smaller re-wiring rate (the largest pairwise comparison p-value<1E-10), suggesting in mammalian PED, evolutionary younger TFs are more likely to change their regulatory targets.

## Discussion

A major goal in biology is to understand the evolution of complex traits, such as morphology and behavior [44]. Pursuit of this goal may be catalyzed by evolutionary models for the molecular substrates, including the coding sequences, the *cis-* regulatory sequences [45], [46], the epigenome, and the transcriptome [26], as well as by evolutionary models for the interaction or regulatory networks of these molecular substrates.

A challenge in modeling these networks lies in the question of how to quantitatively associate gene expression with the “strengths” of *cis-*regulatory regions. From a single species perspective, this challenge has been approached by using sequence rules to predict gene expression under a classification scheme [47] and a regression scheme [48]. Because these schemes both resorted to statistical association, at least in some of their analysis steps, we could not generalize them onto an evolutionary model.

Instead, to link expression and sequence data [49], we introduced the notion of “regulatory state.” Conditional on the regulatory state, the probabilistic forms of both the sequence and the expression data were derived. This enabled a generative probabilistic model for the expression and the sequence data on a phylogenetic tree. We chose to model the expression data in a *soft* way, in the sense that we only modeled the difference of the co-expression patterns between the genes with different regulatory states. This choice suppressed a lot of information from the expression data, but the model seemed to cope well with the noisy nature of the expression data and seemed to capture the essential information on TN in a robust way.

The model can potentially be generalized to treat the combinatorial control of multiple TFs. To do so, the total number of regulatory states should be extended to 2^TF#^, where TF# is the number of TFs in consideration. The evolution of the regulatory states can be modeled as a continuous Markov chain with 2^TF#^ states. The conditional probabilities of the sequence and the expression data should be derived from proper assumptions [50] and recently available information on combinatorial transcriptional regulation [51].

Our evolutionary model predicted that TF-target interactions are more conserved than expression patterns and TF binding events. These are conceptually sensible in several perspectives. First, the modular structure of GRNs allows a small change in the upstream regulators to manifest large changes in the expression of downstream genes without perturbing the *cis-*trans interactions between these genes. For example, the change of the expression pattern or function, such as interactions with other partner proteins, of a master transcriptional regulator may change expression patterns of many downstream genes, while preserving their TF-target relationships by either unperturbed *cis-*regulatory sequences and the DNA binding domain on the TF, or compensatory cis and trans changes that retains regulatory control [51]. This view is consistent the theory of facilitated variation [52]. A recent example is the human transcription factor FoxP2, important for language, on which a small change in the TF (two amino acids, outside DNA binding domain) lead to differential regulation of hundreds of downstream genes [53]. Second, changes of TF binding events do not often lead to TN changes or gene expression changes. The binding sites of a TF may be gained and lost quickly during evolution, but these do not necessarily lead to change of TF regulation of a target gene, as the loss of one site may be compensated by the gain of another site elsewhere in the regulatory region of that gene [11]. Second, regulatory rewiring may happen where the regulator of a gene is switched to another TF in a different species without changing gene expression pattern [54].

The four predicted re-wired Oct4 target genes *OVOL1*, *Id3*, *Ccng1*, and *Rap1gap* were related to development, transcriptional control and signaling, enticing us to speculate that the predicted TN re-wiring events may contribute to interspecies differences during animal development. It turned out that PED is highly conserved among mammals: all progress through the same morphologic stages. Perhaps the most marked difference is the amount of time spent at each stage – a human zygote takes about one day to divide into a 2-cell embryo, whereas a mouse zygote only takes half a day. The predicted re-wiring event on Cyclin G1 (Ccng1), a cyclin regulating cell cycle, may be associated with the interspecies timing difference. This speculation led us to re-examine all of the 64 predicted re-wired target genes, which led to another gene *Orc5l.* Orc5l encodes a subunit of the origin recognition complex, essential for the initiation of the DNA replication in eukaryotic cells. Further experimentation is needed to test whether the differential regulation of Ccng1 and Orc5l could lead to difference in cell cycle time.

Because changes in mRNA quantities may precede changes in protein levels, the inferred conservation and changes in TN may contribute to interspecies differences after the blastocyst embryonic stage. Notable differences include that bovine blastocysts initiate the gastrulation process before implantation, as well as the general formation and functions of the placenta and yolk sac [55]. We therefore mapped the Oct4 targets onto all developmental signaling pathways and identified the three pathways (Tgfb, WNT, mTOR) that contained more than two Oct4 targets [Figure S7]. The three species appear to use Oct4 to regulate conserved components in these three pathways, with a few exceptions. CtBP, a canonical inhibitor of the WNT signal pathway, appears to be regulated by Oct4 in humans and mice, but not in cattle. This might be correlated with the bovine specific role of WNT in placenta development [56].

The positive correlation between the evolutionary age of a TF and the conservation of its regulatory targets has seldom been exploited. The other studies with a similar flavor are on the analyses of the developmental hourglass model [57]. The developmental hourglass refers to the appearance of embryos in related species converges midway (called phylotypic stage) through development and diverges thereafter. Major supports of the model are that genes expressed during the phylotypic stage are both evolutionarily older and more conserved across the genus than those expressed at other stages [58]. PED precedes the phylotypic stage in mammalian development. Still the data showed strong correlation between a TF's evolutionary age and its re-wiring rate. Now that in two separate developmental stages, there are evidences that evolutionarily older TFs serve to regulate more conserved sets of target genes. It would be interesting to see if this relationship checks out in other developmental stages and biological processes.

## Materials and Methods

### Symbols

**Indices**, 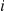: observed species; 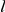: ancestral species; 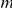: gene. 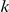: sequence locations; 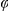: nucleotide, 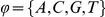; 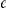: gene clusters. **Observed data**, 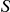: regulatory sequences; 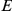: gene expression; 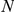: total number of species; 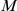: total number of orthologous gene groups; 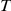: phylogenetic tree; 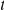: phylogenetic distances (divergence time) between any two nodes. **Data derived from observed data**, 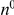: number of background bases in 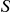; 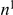: number of TF binding motifs in 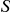; 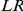: product of likelihood ratio scores of all motifs on 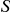; 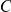: gene cluster index derived from 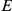; 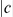: number of gene clusters. **Hidden variables**, 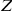: regulatory states. 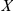: sequence states (background or motif). **Pre-computed parameters**, 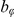: nucleotide frequencies. 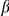: a tuning parameter, representing the weight of the expression data in the likelihood function. **Model parameters**, 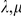: transition probabilities of regulatory states in unit time; 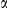: vector of the binary probabilities of the regulatory state in the root node; 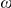: marginal probability of the motif state; 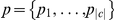: vector of the multinomial probabilities of the cluster index of a target gene. 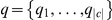: vector of the multinomial probabilities of the cluster index of a non-target gene. 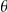: the collection of all model parameters. 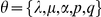.

### Model

#### Data and inference

The data required by this model are genome sequences, gene expression data, a list of candidate TFs and their DNA binding motifs, and estimated divergence time. The model does not require prior information on the exact locations of TFBS or TF-target relationships. The TF-target relationships, *i.e.* the target genes of a TF in every species, are to be inferred by the model.

#### Regulatory states and overall modeling strategy

Let t denote the evolutionary time (Figure 1B). We call the regulatory relationship between a set of TFs (TF_x_) and a target gene 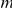 as the regulatory state of this target gene, denoted as 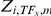, where 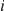 is the species indicator. Here TF_x_ can be one TF or a few interacting TFs. Without loss of generalizability, we suppress the subscript TF_x_ in 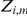. Denote 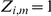 when gene 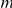 is regulated by TF_x_ in species 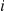, and 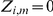 otherwise. Denote 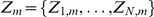, where *N* is the total number of species. The variable 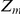 thus indicates whether the regulatory link between the TF and the target gene is conserved or changed over evolutionary time. The general strategy of inferring 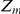 is: if a gene is regulated by a TF, it is likely to contain the binding sites of this TF in its regulatory region and also likely to be co-regulated with other target genes of this TF.

We describe a probabilistic approach to estimate 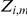. The main idea is to express the joint probability of all sequences 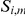 and gene expression data 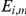 as a product of their conditional probabilities to 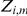. In other words, the likelihood of all sequence and expression data is:
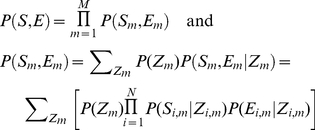
(1)where *N* is the total number of species and *M* is the total number of orthologous gene groups. The model assumption is that in every species, *conditional on* the regulatory state of a target gene, the regulatory sequence of this gene and the expression level of this gene are independent.

This key assumption enabled us to write down the joint probability of the regulatory sequence and the expression level of every gene. We will describe the models for 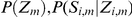 and 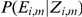 in the following sections.

#### An evolutionary model of the regulatory states

The evolution of the regulatory state 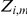 is modeled as a two-state continuous-time Markov chain, with transition probabilities in unit time being λ(0→1) and μ (1→0). The transition probabilities of regulatory states between two species diverged for time *t* is:



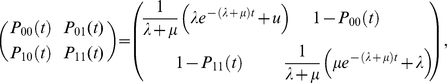
where 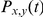 is the transition probability from state *x* to state *y* in time t; *x, y* = {0,1}. Given a phylogenetic tree *T*, the probability of the regulatory state on the leaf node is: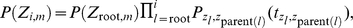 is the parent node of 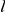; and 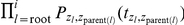 is the product of transition probabilities on the path starting from the root and ending at leaf node 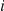. 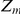 is the collection of the regulatory states in all leaf nodes, descending from the *same* root. It follows that
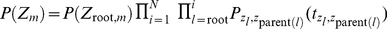
(2)


Where 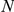 is the total number of observed species (leaf nodes). We introduce another model parameter 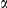, and let 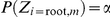.

#### Sequence model

The conditional probability of the regulatory sequence of the target gene 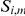 given the regulatory state 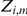 is modeled as a Hidden Markov Model (HMM) [59]. The hidden layer, denoted as 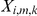, is a two-state (background, motif) Markov chain, where *k* is the index of the state random variable 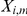. When 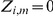, the hidden layer stays at the background state with probability 1, *i.e.* 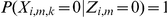 for any *k*. When 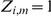, the hidden layer transits between the two states with non-zero probabilities. We denote the marginal probability of an 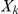 being a motif as 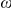, *i.e.* 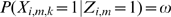. We approximate the HMM by effectively assuming the hidden variables X can be inferred from a motif scanning procedure. Denote 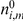 and 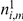 as the number of observed background bases and the number of observed motifs, respectively. 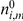 and 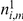 are determined by running a motif scan on 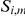. The motif scan calls a segment on 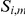 as a motif when the likelihood ratio score of this segment reaches a pre-defined threshold. Under these model assumptions, it follows that



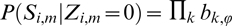
(3.1)where 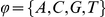, *k* is the index of DNA bases, and 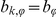 is the background probability of a base being 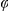. It can be shown that



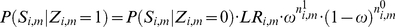
(3.2)where 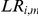 is the product of the likelihood ratio scores of all the reported motifs in 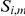. Thus, we explicitly expressed 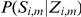 by introducing one extra parameter, 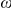, to the model. When there are multiple TFs to be considered, each with its own weight matrix, 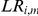 becomes the product of the likelihood ratio scores of all the motifs, reported from the scans of every matrix; 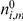 and 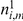 becomes the number of background bases and the total number of motifs for every TF.

#### Expression model

To model the conditional probability of the expression data, we first considered what makes a sensible and quantifiable difference in the expression data between the two regulatory states. We hypothesized that the transcriptional targets of a TF or a set of interacting TFs are likely to co-appear in co-expression modules. We implemented this idea by first clustering the expression data of all the genes. Let 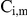 be the cluster index of the m^th^ gene, and 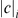 be the total number of clusters. 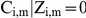 follows a (background) multinomial distribution with parameters 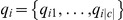, *i.e.*



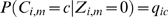
(4.1)where 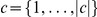. When 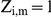, the subset of genes, which are transcriptionally regulated by the TF or the set of interacting TFs, would tend to concentrate in a subset of the clusters. Thus, 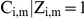 follows another multinomial distribution with parameters 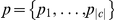, *i.e.*



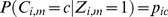
(4.2).

Thus, by inserting probabilities (2) - (4.2) into (1), we derived the complete likelihood of all data.

#### Weighing sequence and expression data

The likelihood model in (1), although it is completely specified, assumes equal weights of the sequence and the expression data. We further introduced a tuning parameter 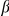 to adjust the relative weights of the two data types. Thus, the model becomes:

and



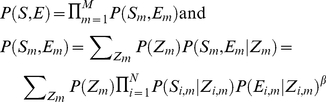
(5).

The larger 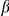 is, the more weight is given to the expression data.

#### Model fitting

We developed an estimation-maximization (EM) algorithm to estimate the model parameters. The E-step estimates 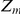, and the M-step maximizes 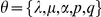. Denote 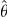 as the maximum likelihood estimator (MLE) of 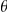. The complete E-M algorithm is available in Text S2.

#### Model inference

To identify the most likely regulatory states of all the genes in an orthologous group 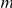, we use 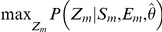 to simultaneously estimate 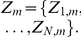


### Methods

#### Yeast data

Gene expression data of *C. albicans* and *S. cerevisiae* were downloaded from the online supplementary material of Ihmels *et al.* [29]. Gene expression data of *C. glabrata* were collected from GEO (GSE6626, GSE6582, and GSE6058). The map of orthologous genes was obtained from the Yeast Gene Order Browser v.3 [60]. The genes that do not have orthologs in all the three yeast species were eliminated. The remaining 51 mitochondrial ribosomal protein (MRP), 58 rRNA, and 73 stress-related (STR) orthologous gene triplets were used in the TN evolution analysis. The 300bp upstream sequences to the TSSs were obtained from genome databases (www.yeastgenome.org, www.candidagenome.org, wolfe.gen.tcd.ie/ygob/Cglabrata_sequence.fsa (v.3)). We used the phylogenetic topology and the evolutionary distances estimated by Tuch *et al.* [61].

#### Preprocessing yeast data

The 182 genes in each species were independently clustered by their expression data, using k-means clustering with 3 centers. An enriched sequence motif was identified by applying MEME [30] to the upstream sequences of *C. albicans, S. cerevisiae,* and *C. glabrata* rRNA genes, as well as *C. albicans* MRP genes (Figure S3-B New Motif).

#### Comparing model prediction with Ihmels' theory

Ihmels *et al.* suggested that the rRNA genes in all three yeast species and all the MRP genes in *C. albicans* are regulated by the conserved transcription factor TFa; moreover, none of the STR genes in any species and none of the MRP genes in any anaerobic species is regulated by TFa (Table S2) [29]. Our prediction is compared with this theory. For every gene in each species, the TN evolution model predicts whether it is regulated by TFa. We assumed a prediction to be correct if it matched Ihmels' theory, by checking Table S2. By matching Ihmels' theory we meant consistent to the notion that the MRP genes have been re-wired but other genes are not. For example, if a MRP gene in *C. albicans* was predicted to be a target of TFa, this prediction was considered correct. The prediction accuracy of the model was defined as the ratio between the total number of correct predictions and the total number of genes in the three species.

#### Mammalian data

The gene expression data of PED in humans, mice, and cattle were obtained from Xie *et al.* (GEO: GSE18290, GSE18319) [8]. The orthologous gene map was obtained from Xie *et al.* [8]. The upstream sequence of a gene was defined as the 20 k bp sequence flanking the TSS. These sequences were obtained from UCSC Genome Browser. The Oct4 motif was obtained from Chen *et al.* [37]. The phylogenetic distances of the three species were obtained from [62].

#### Preprocessing mammalian data

The orthologous gene triplets were filtered out, if none of the triplets had a clear change of expression levels (coefficient of variation >1.26) during PED. After the filtering, 1,509 orthologous gene triplets were passed onto clustering analysis.

The clustering of genes was performed independently within each of the three species, using a recently developed clustering method based on a Dirichlet Process [51]. Without requiring a pre-determined cluster number, this clustering algorithm automatically determines the optimum cluster number supported by the data. After removing singletons in the clustering result, 1,489 genes formed 27, 22, and 26 clusters in humans, mice, and cattle, respectively.

The probabilities 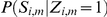 and 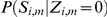 were computed for every 500bp sliding window on the 20 k bp sequence of a gene. The sliding window with the largest 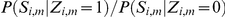 was selected as the representative window of the 20 k bp sequence. The 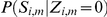 and 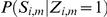 from this representative window were used as the probabilities in (3.1) and (3.2) for the 20 k bp sequence [Figure S4].

#### Choosing the weight (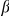) parameter in PED analysis

Having a weight (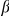) parameter in the likelihood function is a commonly used approach to hybridize probability functions of heterogeneous data types. In our case the likelihood calculated from sequence data and the likelihood calculated from expression data can be in different scales. The weight serves as a scaling adjustment to make contributions from both sides comparable. In the analysis of PED data, we assumed that real data were very noisy and therefore were most similar to the “weak sequence signal and weak expression signal” case in the simulation. We directly assigned the weight (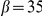) which seems to work fine in simulation on “both weak” data in the analysis of real data. We checked two other cases (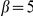 and 50) and found in those cases the model predictions were the same as the model predictions when ignoring the expression and ignoring the sequence data, respectively, as expected.

## Supporting Information

Figure S1 Histogram of human-mouse gene expression correlations. The human-mouse orthologous genes were identified by best blast bi-directional hits (BBH). Gene expression data were obtained from human and mouse gene atlas project, which used gene-chip microarrays to assay various tissues. In gene atlas data contained a total of 28 human-mouse matched tissues, and a total of 2,534 human-mouse BBH gene pairs on the microarrays. For each orthologous gene pair, a Pearson correlation ρ of their two-species gene expression was calculated, based on their expression levels in 28 matched tissues. 39.7% of the BBH orthologous pairs are negatively correlated; 64.2% had a correlation <0.1, and 91.2% had a correlation <0.6, suggesting large interspecies expression differences.
(TIF)

Figure S2Prediction accuracies for simulated datasets. The predication accuracy was plotted against the relative weight of the expression data (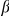). Training and testing datasets were separate datasets. The prediction accuracies on training and testing datasets are almost identical, rendering the accuracy curves to overlap.(EPS)

Figure S3 Rewiring of TNs among three yeast species. (A) Clustering of gene expression data in each species. The functional gene groups, including mitochondria protein genes (MRP), rRNA genes (rRNA), and stress response genes (STR), are correlated with gene clusters. (B) Prediction accuracy of regulatory relationships using the new motif (blue) and using the Ihmels *et al.* reported motif (red).
(TIF)

Figure S4 Preparing the sequence and the expression data for analysis by the evolutionary model of TNs.
(EPS)

Figure S5 Binding site turnover. Rex box indicates the predicted transcription factor binding sites (TFBS) in human (A) and mouse (B–D). Nucleotide sequences in red on top of the red boxes represent common ancestral sequence reconstructed by parsimonious reconstruction. Mouse and rat experienced a deletion event that removed 4 bp out of the 8 bp TFBS, probably causing a death of the TFBS (A). Another 2 bp deletion from ACAgtACCGTG (ancestral) into ACAACCGTG gave birth to a murine specific binding site (B). Species-specific insertion (C) and mutation (D) could also lead to births of TFBSs.
(TIF)

Figure S6 Numbers of predicted conserved Oct4 target genes, protein-protein interactions, and gene regulatory relationships.
(TIF)

Figure S7 Conserved and alternatively regulated signaling pathway components. Canonical components of TGFβ, WNT, and mTOR pathways are shown. A gene in white is not a transcriptional target of OCT4-SOX2. A gene is colored with blue, red, or green when its human, mouse, or bovine ortholog is a transcriptional target of OCT4-SOX2, respectively. A gene with two or three colors is a target in two or three species. For example, c-Myc is colored blue, red, and green, and its orthologs in all three species are OCT4-SOX2 targets.
(TIF)

Figure S8 Model-inferred Oct4 target genes and the protein-protein interactions among the gene products.
(TIF)

Table S1 Evolutionary models for gene regulatory sequence, gene expression and using phylogenetic information.
(PDF)

Table S2 The regulatory relationship between TF_a_ and three sets of genes in three species. The number of genes found in each set is given in parentheses.
(PDF)

Text S1 Supplementary Data. Description of simulation study data generation.
(PDF)

Text S2 Supplementary Methods. Parameters for simulation datasets, methods to measure prediction accuracy and description of E-M algorithm for parameter estimation.
(PDF)
