# Supplementary figures and images for: Towards an Evolutionary Model of Transcription Networks
Source: PLoS Comput Biol. 2011 Jun 9;7(6):e1002064. doi: 10.1371/journal.pcbi.1002064 (PMC3111474; doi:10.1371/journal.pcbi.1002064)

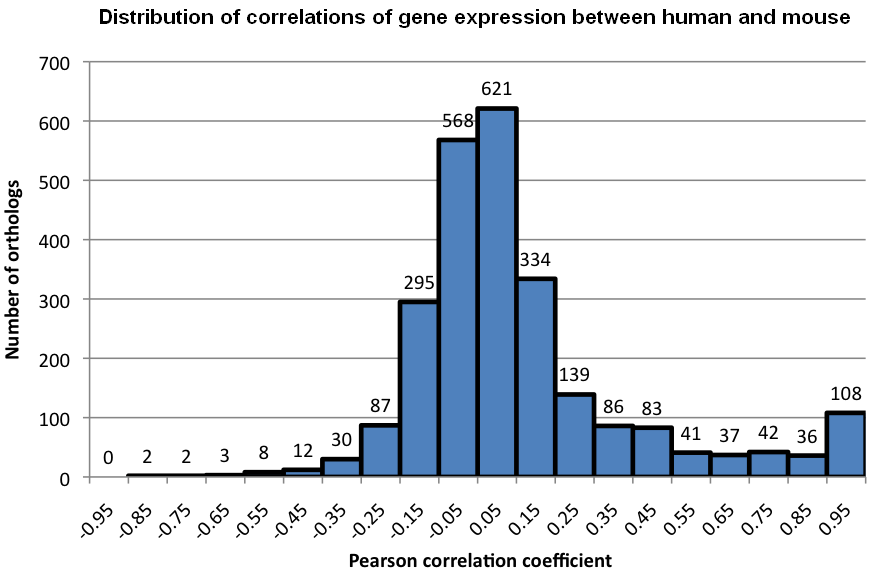

Supplement: Figure S1 — Histogram of human-mouse gene expression correlations. The human-mouse orthologous genes were identified by best blast bi-directional hits (BBH). Gene expression data were obtained from human and mouse gene atlas project, which used gene-chip microarrays to assay various tissues. In gene atlas data contained a total of 28 human-mouse matched tissues, and a total of 2,534 human-mouse BBH gene pairs on the microarrays. For each orthologous gene pair, a Pearson correlation ρ of their two-species gene expression was calculated, based on their expression levels in 28 matched tissues. 39.7% of the BBH orthologous pairs are negatively correlated; 64.2% had a correlation <0.1, and 91.2% had a correlation <0.6, suggesting large interspecies expression differences. (TIF) [file pcbi.1002064.s001.tif]

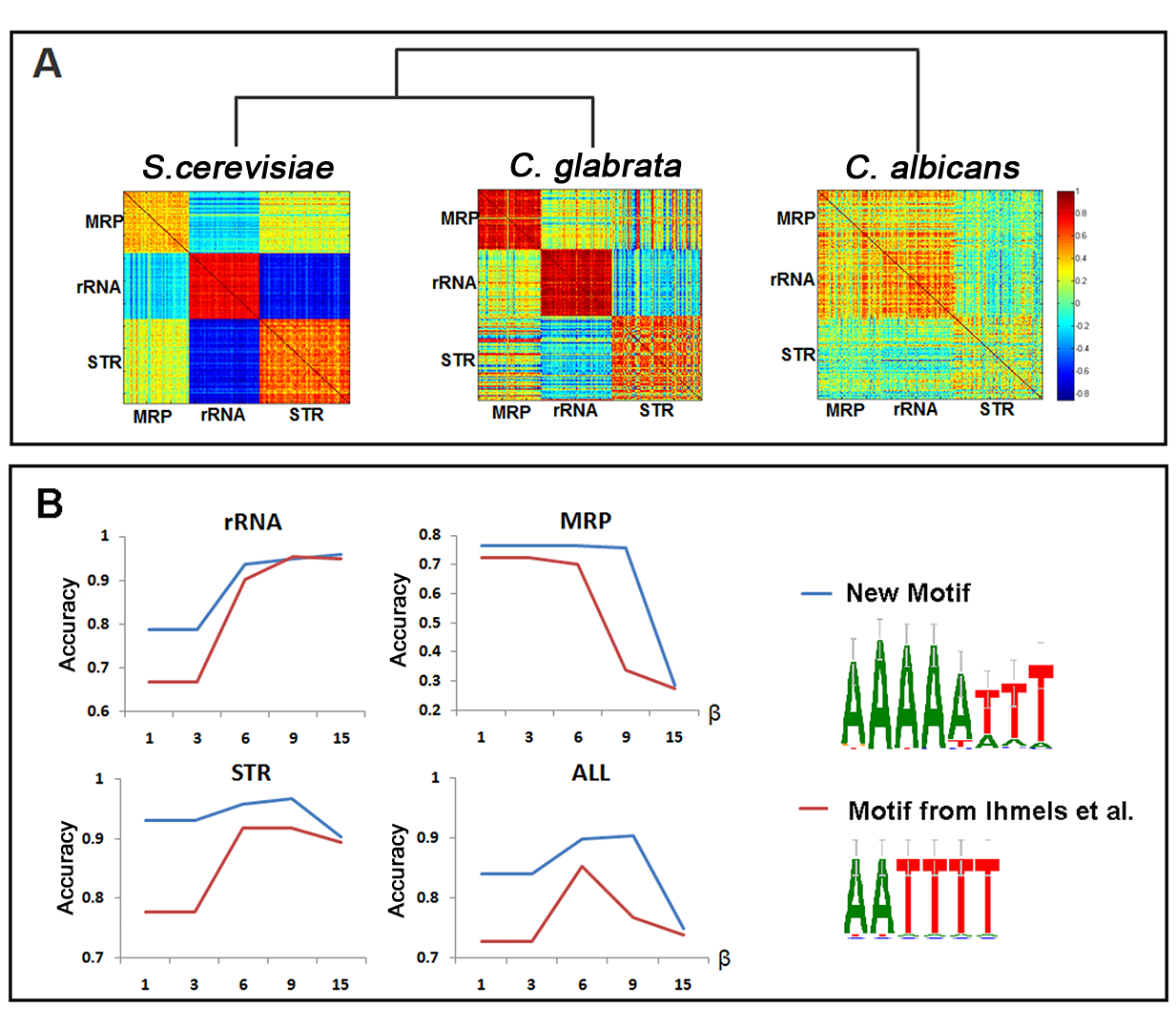

Supplement: Figure S3 — Rewiring of TNs among three yeast species. (A) Clustering of gene expression data in each species. The functional gene groups, including mitochondria protein genes (MRP), rRNA genes (rRNA), and stress response genes (STR), are correlated with gene clusters. (B) Prediction accuracy of regulatory relationships using the new motif (blue) and using the Ihmels et al. reported motif (red). (TIF) [file pcbi.1002064.s003.tif]

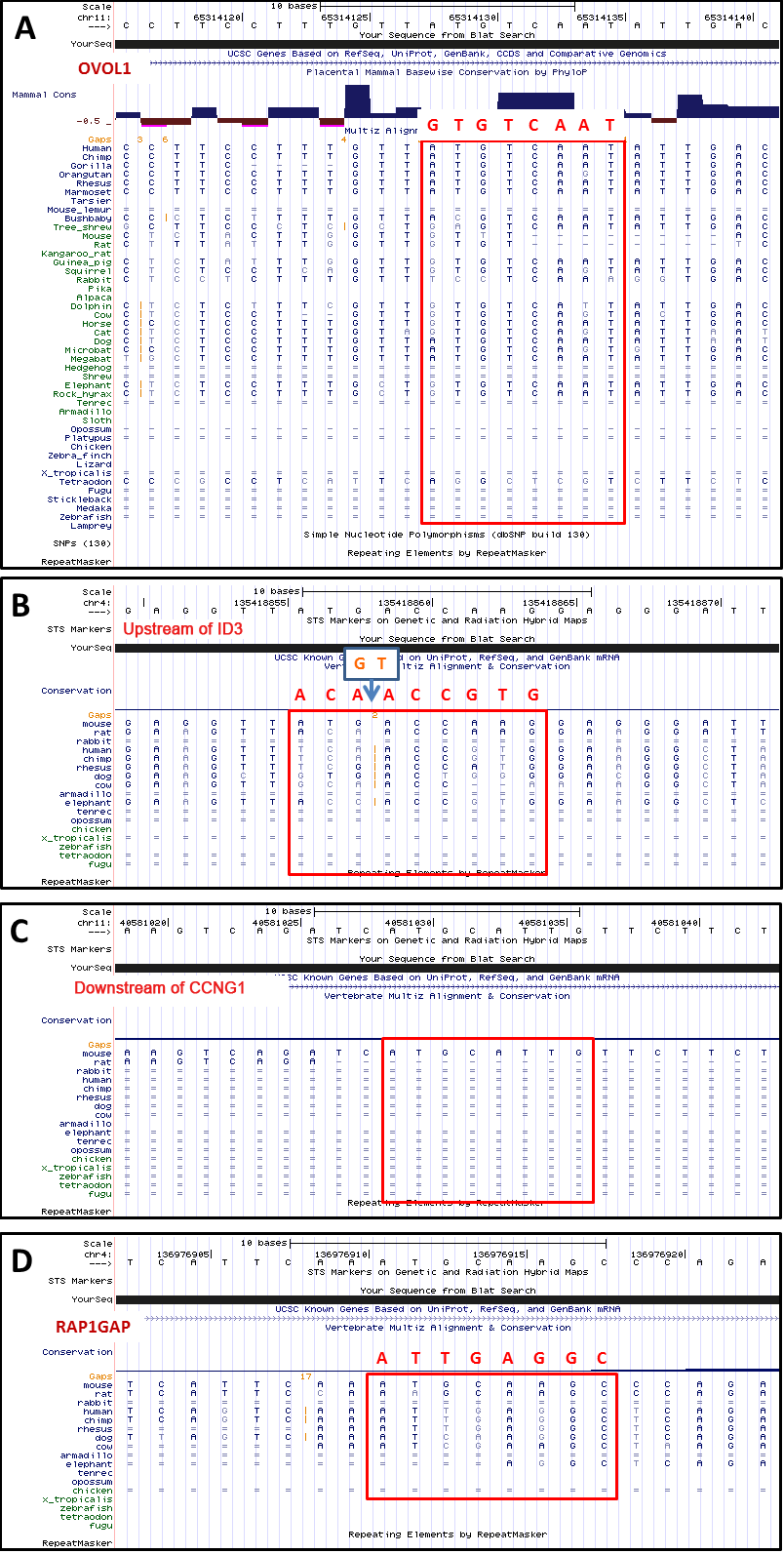

Supplement: Figure S5 — Binding site turnover. Rex box indicates the predicted transcription factor binding sites (TFBS) in human (A) and mouse (B–D). Nucleotide sequences in red on top of the red boxes represent common ancestral sequence reconstructed by parsimonious reconstruction. Mouse and rat experienced a deletion event that removed 4 bp out of the 8 bp TFBS, probably causing a death of the TFBS (A). Another 2 bp deletion from ACAgtACCGTG (ancestral) into ACAACCGTG gave birth to a murine specific binding site (B). Species-specific insertion (C) and mutation (D) could also lead to births of TFBSs. (TIF) [file pcbi.1002064.s005.tif]

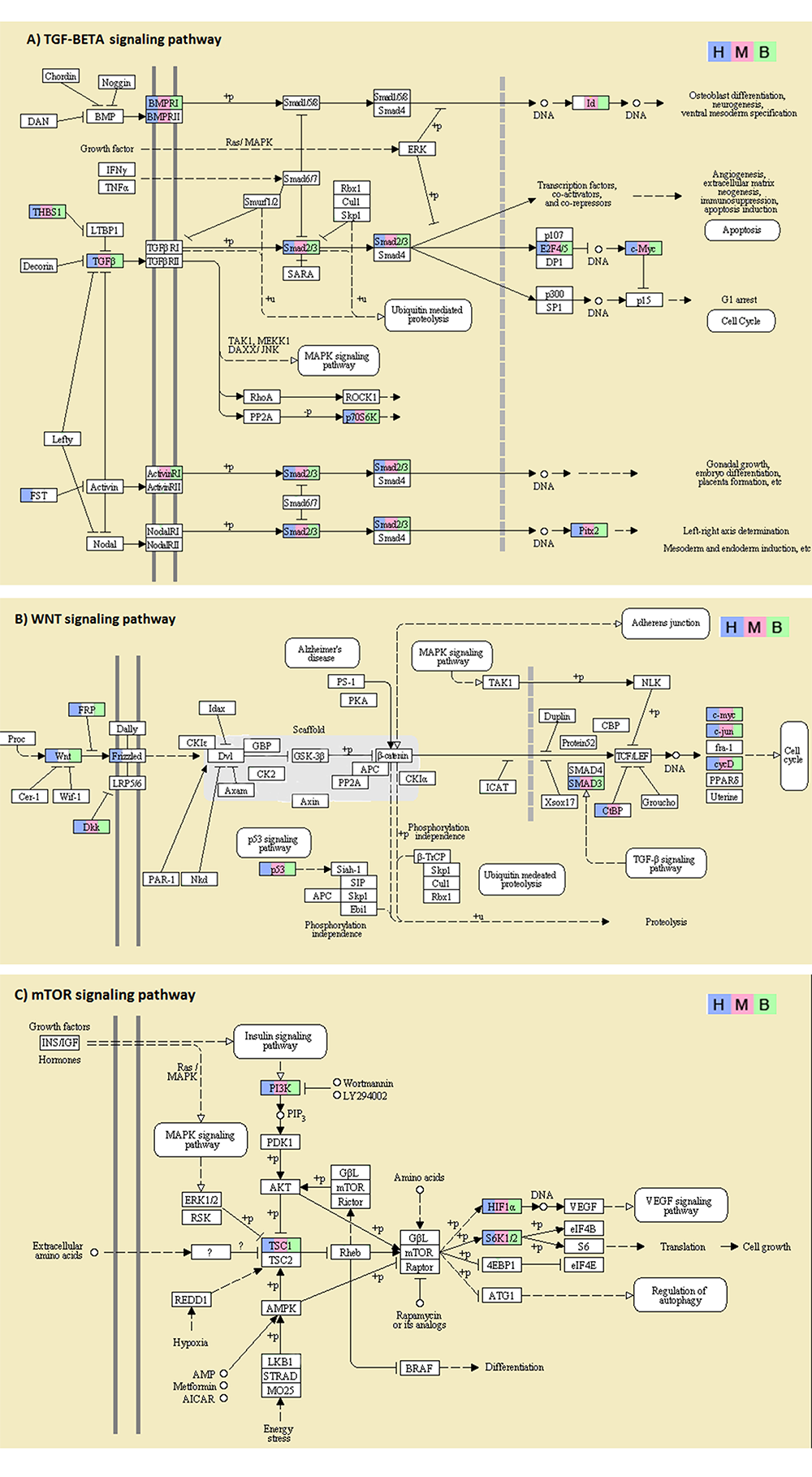

Supplement: Figure S7 — Conserved and alternatively regulated signaling pathway components. Canonical components of TGFβ, WNT, and mTOR pathways are shown. A gene in white is not a transcriptional target of OCT4-SOX2. A gene is colored with blue, red, or green when its human, mouse, or bovine ortholog is a transcriptional target of OCT4-SOX2, respectively. A gene with two or three colors is a target in two or three species. For example, c-Myc is colored blue, red, and green, and its orthologs in all three species are OCT4-SOX2 targets. (TIF) [file pcbi.1002064.s007.tif]

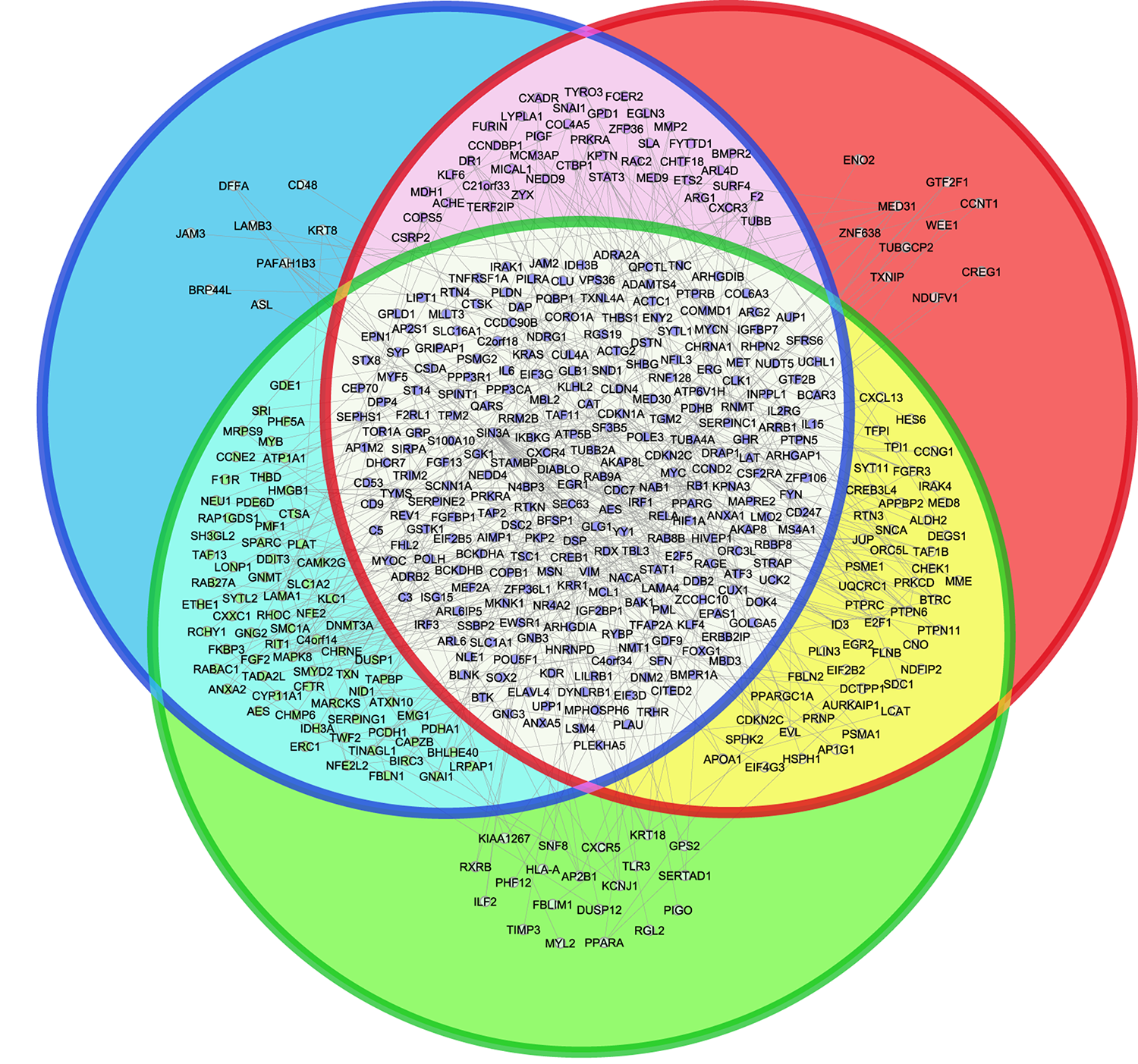

Supplement: Figure S8 — Model-inferred Oct4 target genes and the protein-protein interactions among the gene products. (TIF) [file pcbi.1002064.s008.tif]
