# Supplementary material for: Towards an Evolutionary Model of Transcription Networks
Source: PLoS Comput Biol. 2011 Jun 9;7(6):e1002064. doi: 10.1371/journal.pcbi.1002064 (PMC3111474; doi:10.1371/journal.pcbi.1002064)
Supplement: Table S1 — Evolutionary models for gene regulatory sequence, gene expression and using phylogenetic information. (PDF) [file pcbi.1002064.s009.pdf]

Table S1: Evolutionary models for gene regulatory sequence (S), gene expression (E) and using phylogenetic information (P).

Model	Components			Applications	Species
**MAFIA[1]**	P	S	-	Evolution of binding sites	Drosophila
**EMMA[2]**	P*	S	-	CRM alignment and prediction	Drosophila
**Kimono[3]**	-	S	E	Clustering of promoter and expression	Simulation
**MODEM[4]**	-	S	E	Identifying target genes of a transcription factor	Yeast
**REDUCE[5]**	-	S	E	Motif prediction	Yeast
**DCA[6]**	P	-	E	Identifying conserved and diverged co-expression patterns	Yeasts
**SCSC[7]**	P*	-	E	Identifying conserved and diverged co-expression patterns	Mammals
**Khaitovich[8]**	P	-	E	Neutral evolution of gene expression	Primates
**Xie [this paper]**	P	S	E	Evolution of gene regulatory networks	Yeasts, Mammals

* Only applied to two species.

1. Majoros WH, Ohler U (2010) Modeling the Evolution of Regulatory Elements by Simultaneous Detection and Alignment with Phylogenetic Pair HMMs. PLoS Comput Biol 6: e1001037.
2. He X, Ling X, Sinha S (2009) Alignment and Prediction of <italic>cis</italic>-Regulatory Modules Based on a Probabilistic Model of Evolution. PLoS Comput Biol 5: e1000299.
3. Holmes I, Bruno W (2000) Finding regulatory elements using joint likelihoods for sequence and expression profile data. Proc Int Conf Intell Syst Mol Biol 8: 202-210.
4. Wang W, Cherry JM, Botstein D, Li H (2002) A systematic approach to reconstructing transcription networks in Saccharomycescerevisiae. Proceedings of the National Academy of Sciences of the United States of America 99: 16893-16898.
5. Bussemaker HJ, Li H, Siggia ED (2001) Regulatory element detection using correlation with expression. Nat Genet 27: 167-174.
6. Ihmels J, Bergmann S, Berman J, Barkai N (2005) Comparative gene expression analysis by differential clustering approach: application to the Candida albicans transcription program. PLoS genetics 1: e39.
7. Cai J, Xie D, Fan Z, Chipperfield H, Marden J, et al. (2010) Modeling Co-Expression across Species for Complex Traits: Insights to the Difference of Human and Mouse Embryonic Stem Cells. PLoS Comput Biol 6: e1000707.
8. Khaitovich P, Paabo S, Weiss G (2005) Toward a Neutral Evolutionary Model of Gene Expression. Genetics 170: 929-939.
