# Supplementary material for: Towards an Evolutionary Model of Transcription Networks
Source: PLoS Comput Biol. 2011 Jun 9;7(6):e1002064. doi: 10.1371/journal.pcbi.1002064 (PMC3111474; doi:10.1371/journal.pcbi.1002064)
Supplement: Table S2 — The regulatory relationship between TFa and three sets of genes in three species. The number of genes found in each set is given in parentheses. (PDF) [file pcbi.1002064.s010.pdf]

Table S2: The regulatory relationship between TFa and three sets of genes in three species. The number of genes found in each set is given in parentheses.

Phenotype	Genes / Species	Regulated by TFa		
		rRNA (58)	STR (73)	MRP (51)
anaerobic	*S. cerevisiae*	No	Yes	No
	C. glabrata	No	Yes	No
aerobic	*C. albicans*	No	Yes	Yes
