# Supplementary material for: Towards an Evolutionary Model of Transcription Networks
Source: PLoS Comput Biol. 2011 Jun 9;7(6):e1002064. doi: 10.1371/journal.pcbi.1002064 (PMC3111474; doi:10.1371/journal.pcbi.1002064)
Supplement: Text S1 — Supplementary Data. Description of simulation study data generation. (PDF) [file pcbi.1002064.s011.pdf]

Text S1 Supplementary Data

Simulation study

We simulated a series of six synthetic datasets to test the model. The data generation steps are as follows. First, we designed a phylogenetic tree T with three leaf nodes and designated the evolutionary parameters, including the divergence time t and the transition probabilities λ, μ of regulatory states in a unit time. Second, we simulated 1000 orthologous gene groups. Each gene group was composed of a triplet of genes in the observed species. The regulatory state of each gene in each species was simulated from the continuous time Markov chain. Third, we proceeded to simulate the regulatory sequence for each gene. We decided on the number of TFBSs in this sequence by drawing from a $Poisson$ distribution. When the regulatory state was 1, we drew from $Poisson(\lambda_1)$; otherwise we drew from $Poisson(\lambda_0)$. We used different λ_1 and λ_0 to generate multiple simulated datasets. Next, we generated the drawn number of TFBSs by sampling from the product's multinomial distribution defined by the OCT4 position-specific weight matrix (PWM). These TFBSs were inserted into a background sequence, and the full sequence was truncated into length 1000bp. Finally, we designated the total number of clusters as 5. We simulated the cluster indicator for each gene according to multinomial distributions p and q. Different p and q were used in the different simulation datasets.

The 6 simulated datasets had different signal and noise levels. Here the signal refers to the information that could be utilized by our model for predicting TN structure. The first simulated dataset contained strong signals in both the sequence and the expression data. On this dataset the model achieved 85% and above prediction accuracies (Figure S2 "Both strong"). When we tested β from small (0.05) to large (10 -100) values, the prediction accuracy first increased, and peaked at around $\beta = 1$, and then decreased. This is consistent with our expectation because when β is very small (large), the model relies almost exclusively on the sequence (expression) data, which should not be as good as when the signals from both data types are utilized. The shape of the prediction accuracy curve suggests that the model was capable of taking real advantage of having two data types. We then hypothesized that the prediction accuracy when β is small (large) would be similar to the prediction accuracy when the model is applied to a dataset with a similar signal level in sequence (expression) data and no signal at all in expression (sequence) data. To test this hypothesis, we simulated two other datasets, which followed the same simulation procedure for the sequence (expression) data but randomly generated the expression (sequence) data. The model's prediction accuracies behaved as expected on these two datasets (Figure S2, "Sequence only" and "Expression only"). We then further challenged the model by adding a little signal to the expression data of the "Sequence only" dataset, which led to improved prediction accuracy in the usual range of β (0.5 - 3) (Figure S2, "StrongSeq WeakExp"). Similarly, we found adding a little signal to the sequence data of the "Expression only" dataset led to improved prediction

accuracy (Figure S2, “StrongExp WeakSeq”). Finally, as a negative control, we generated a dataset with little signals in both the sequence and the expression data, which led to the smallest prediction accuracies of all tested β values (Figure S2, “Both weak”). These simulation results suggest the TN evolution model successfully utilized both the sequence and the expression data for predicting the regulatory relationships in multiple species.

To assess potential model overfitting, we performed 5-fold cross-validation on each of the six simulated datasets (Figure S2). The model performances on training data and testing data of all six simulations were very similar, suggesting that in various simulated situations, including the combinations of strong or weak sequencing signals to strong or weak coexpression signals, the model would not overfit, regardless of the choice of β.
