# Supplementary material for: Towards an Evolutionary Model of Transcription Networks
Source: PLoS Comput Biol. 2011 Jun 9;7(6):e1002064. doi: 10.1371/journal.pcbi.1002064 (PMC3111474; doi:10.1371/journal.pcbi.1002064)
Supplement: Text S2 — Supplementary Methods. Parameters for simulation datasets, methods to measure prediction accuracy and description of E-M algorithm for parameter estimation. (PDF) [file pcbi.1002064.s012.pdf]

Text S2 Supplementary Methods

Simulation

Six datasets were simulated with different signal and noise levels in the sequence and in the expression data. The parameters used for simulation are as follows. Both strong: $\lambda_0 = 0.5$, $\lambda_1 = 2$, $p = \{0.2, 0.2, 0.2, 0.2, 0.2\}$, $q = \{0.9, 0.025, 0.025, 0.025, 0.025\}$.

Sequence only: $\lambda_0 = 0.5$, $\lambda_1 = 2$, $p = \{0.2, 0.2, 0.2, 0.2, 0.2\}$, $q = \{0.2, 0.2, 0.2, 0.2, 0.2\}$.

Expression only: $\lambda_0 = 1.5$, $\lambda_1 = 1.5$, $p = \{0.2, 0.2, 0.2, 0.2, 0.2\}$, $q = \{0.9, 0.025, 0.025, 0.025, 0.025\}$.

StrongSeq WeakExp: $\lambda_0 = 0.5$, $\lambda_1 = 2$, $p = \{0.2, 0.2, 0.2, 0.2, 0.2\}$, $\mathrm{q} = \{0.6, 0.1, 0.1, 0.1, 0.1\}$.

StrongExp WeakSeq: $\lambda_0 = 1$, $\lambda_1 = 2$, $p = \{0.2, 0.2, 0.2, 0.2, 0.2\}$, $q = \{0.9, 0.025, 0.025, 0.025, 0.025\}$.

Both weak: $\lambda_0 = 1$, $\lambda_1 = 2$, $p = \{0.2, 0.2, 0.2, 0.2, 0.2\}$, $\mathrm{q} = \{0.6, 0.1, 0.1, 0.1, 0.1\}$.

Measuring prediction accuracy in simulation studies

The prediction accuracy of the model was measured by the proportion of orthologous groups with correctly predicted regulatory states. An orthologous gene triplet was noted correctly predicted only when all three predicted regulatory states were correct. In other words, if a gene triplet with regulatory states (1,0,0) was predicted as (1,1,0), this prediction on this triplet would be judged as wrong, even though two of the three predicted regulatory states were correct.

E-M algorithm for model inference

Without loss of generalizability, we assume there are three species in consideration, allowing a total of $2^3 = 8$ possible configurations of regulatory states. The probability for each configuration (R) is:

$$R_{m,1}\text{: } P\left(S_m, C_m, Z_{1,m} = 0, Z_{2,m} = 0, Z_{3,m} = 0 \middle| \theta\right) = (1-\alpha)^3 P\left(Tree_{Z_{1,m}=0, Z_{2,m}=0, Z_{3,m}=0}\right) P(S_{1,m}|Z_{1,m} = 0) P(S_{2,m}|Z_{2,m} = 0) P(S_{3,m}|Z_{3,m} = 0) (q_{1c,m} q_{2c,m} q_{3c,m})^{\beta}$$

$$R_{m,2}\text{: } P\left(S_m, C_m, Z_{1,m} = 0, Z_{2,m} = 0, Z_{3,m} = 1 \middle| \theta\right) = \alpha(1-\alpha)^2 P\left(Tree_{Z_{1,m}=0, Z_{2,m}=0, Z_{3,m}=1}\right) P\left(S_{1,m} \middle| Z_{1,m} = 0\right) P\left(S_{2,m} \middle| Z_{2,m} = 0\right) P\left(S_{3,m} \middle| Z_{3,m} = 0\right) LR_{3,m} \omega^{n_{3,m}^1} (1-\omega)^{n_{3,m}^0} (q_{1c,m} q_{2c,m} p_{3c,m})^{\beta}$$

$$R_{m,3}\text{: } P\left(S_m, C_m, Z_{1,m} = 0, Z_{2,m} = 1, Z_{3,m} = 0 \middle| \theta\right) = \alpha(1-\alpha)^2 P\left(Tree_{Z=Z_{1,m}=0, Z_{2,m}=1, Z_{3,m}=0}\right) P\left(S_{1,m} \middle| Z_{1,m} = 0\right) P\left(S_{2,m} \middle| Z_{2,m} = 0\right) P\left(S_{3,m} \middle| Z_{3,m} = 0\right) LR_{2,m} \omega^{n_{2,m}^1} (1-\omega)^{n_{2,m}^0} (q_{1c,m} p_{2c,m} q_{3c,m})^{\beta}$$

$R_{m,4}$: $P\big(S_m, C_m, Z_{1,m} = 0, Z_{2,m} = 1, Z_{3,m} = 1\big|\theta\big)$
$= \alpha^2(1$
$- \alpha)P\big(Tree_{Z_{1,m}=0,Z_{2,m}=1,Z_{3,m}=1}\big)P\big(S_{1,m}\big|Z_{1,m} = 0\big)P\big(S_{2,m}\big|Z_{2,m} = 0\big)P\big(S_{3,m}\big|Z_{3,m} = 0\big)LR_{2,m}\omega^{n_{2,m}^1}(1$
$- \omega)^{n_{2,m}^0}LR_{3,m}\omega^{n_{3,m}^1}(1-\omega)^{n_{3,m}^0}(q_{1c,m}p_{2c,m}p_{3c,m})^\beta$

$R_{m,5}$: $P\big(S_m, C_m, Z_{1,m} = 1, Z_{2,m} = 0, Z_{3,m} = 0\big|\theta\big)$
$= \alpha(1$
$- \alpha)^2P\big(Tree_{Z_{1,m}=1,Z_{2,m}=0,Z_{3,m}=0}\big)P\big(S_{1,m}\big|Z_{1,m} = 0\big)P\big(S_{2,m}\big|Z_{2,m} = 0\big)P\big(S_{3,m}\big|Z_{3,m} = 0\big)LR_{1,m}\omega^{n_{1,m}^1}(1$
$- \omega)^{n_{1,m}^0}(p_{1c,m}q_{2c,m}q_{3c,m})^\beta$

$R_{m,6}$: $P\big(S_m, C_m, Z_{1,m} = 1, Z_{2,m} = 0, Z_{3,m} = 1\big|\theta\big)$
$= \alpha^2(1$
$- \alpha)P\big(Tree_{Z_{1,m}=1,Z_{2,m}=0,Z_{3,m}=1}\big)P\big(S_{1,m}\big|Z_{1,m} = 0\big)P\big(S_{2,m}\big|Z_{2,m} = 0\big)P\big(S_{3,m}\big|Z_{3,m} = 0\big)LR_{1,m}\omega^{n_{1,m}^1}(1$
$- \omega)^{n_{1,m}^0}LR_{3,m}\omega^{n_{3,m}^1}(1-\omega)^{n_{3,m}^0}(p_{1c,m}q_{2c,m}p_{3c,m})^\beta$

$R_{m,7}$: $P\big(S_m, C_m, Z_{1,m} = 1, Z_{2,m} = 1, Z_{3,m} = 0\big|\theta\big)$
$= \alpha^2(1$
$- \alpha)P\big(Tree_{Z_{1,m}=1,Z_{2,m}=1,Z_{3,m}=0}\big)P\big(S_{1,m}\big|Z_{1,m} = 0\big)P\big(S_{2,m}\big|Z_{2,m} = 0\big)P\big(S_{3,m}\big|Z_{3,m} = 0\big)LR_{1,m}\omega^{n_{1,m}^1}(1$
$- \omega)^{n_{1,m}^0}LR_{2,m}\omega^{n_{2,m}^1}(1-\omega)^{n_{2,m}^0}(p_{1c,m}p_{2c,m}q_{3c,m})^\beta$

$R_{m,8}$: $P\big(S_m, C_m, Z_{1,m} = 1, Z_{2,m} = 1, Z_{3,m} = 1\big|\theta\big)$
$= \alpha^2P\big(Tree_{Z_{1,m}=1,Z_{2,m}=1,Z_{3,m}=1}\big)P\big(S_{1,m}\big|Z_{1,m} = 0\big)P\big(S_{2,m}\big|Z_{2,m} = 0\big)P\big(S_{3,m}\big|Z_{3,m} = 0\big)LR_{1,m}\omega^{n_{1,m}^1}(1$
$- \omega)^{n_{1,m}^0}LR_{2,m}\omega^{n_{2,m}^1}(1-\omega)^{n_{2,m}^0}LR_{3,m}\omega^{n_{3,m}^1}(1-\omega)^{n_{3,m}^0}(p_{1c,m}p_{2c,m}p_{3c,m})^\beta$

Let

$$r_{m,a} = \frac{R_{m,a}}{\sum_{j=1}^{8} R_{m,j}}$$

The expectation of likelihood (Q-function) is:

$$Q(\theta|\theta^t) = \sum_{m=1}^{M}\sum_{j=1}^{8} r_{m,j}\log(R_{m,j})$$

We have the constraint: $\sum_{k=1}^{|C|_i} p_{ic} = 1$. Let t be the Lagrange multiplier, $\hat{Q} = Q + t\left(\sum_{k=1}^{|C|_i} p_{ic} - 1\right)$.

To estimate α:

$$\frac{\partial\hat{Q}}{\partial\alpha} = \frac{\partial Q}{\partial\alpha} = \sum_{m=1}^{M}\Big(\frac{-3r_{m,1}}{1-\alpha} + \frac{r_{m,2}}{\alpha} - \frac{2r_{m,2}}{1-\alpha} + \frac{r_{m,3}}{\alpha} - \frac{2r_{m,3}}{1-\alpha} + \frac{2r_{m,4}}{\alpha} - \frac{r_{m,4}}{1-\alpha} + \frac{r_{m,5}}{\alpha} - \frac{2r_{m,5}}{1-\alpha} + \frac{2r_{m,6}}{\alpha} - \frac{r_{m,6}}{1-\alpha} + \frac{r_{m,7}}{\alpha} - \frac{2r_{m,7}}{1-\alpha} + \frac{3r_{m,8}}{\alpha}\Big) = 0$$

$$\hat{\alpha} = \sum_{m=1}^{M}\frac{r_{m,2} + r_{m,3} + 2r_{m,4} + r_{m,5} + 2r_{m,6} + 2r_{m,7} + 3r_{m,8}}{3}$$

To estimate ω:

$$\frac{\partial \hat{Q}}{\partial \omega} = \frac{\partial Q}{\partial \omega} = \sum_{m=1}^{M} \frac{r_{m,2} n_{3,m}^1}{\omega} - \frac{r_{m,2} n_{3,m}^0}{1-\omega} + \frac{r_{m,3} n_{2,m}^1}{\omega} - \frac{r_{m,3} n_{2,m}^0}{1-\omega} + \frac{r_{m,4} n_{2,m}^1 + r_{m,4} n_{3,m}^1}{\omega} - \frac{r_{m,4} n_{2,m}^0 + r_{m,4} n_{3,m}^0}{1-\omega}$$
$$+ \frac{r_{m,5} n_{1,m}^1}{\omega} - \frac{r_{m,5} n_{1,m}^0}{1-\omega} + \frac{r_{m,6} n_{1,m}^1 + r_{m,6} n_{3,m}^1}{\omega} - \frac{r_{m,6} n_{1,m}^0 + r_{m,6} n_{3,m}^0}{1-\omega} + \frac{r_{m,7} n_{1,m}^1 + r_{m,7} n_{2,m}^1}{\omega}$$
$$- \frac{r_{m,7} n_{1,m}^0 + r_{m,7} n_{2,m}^0}{1-\omega} + \frac{r_{m,8} n_{1,m}^1 + r_{m,8} n_{2,m}^1 + r_{m,8} n_{3,m}^1}{\omega} - \frac{r_{m,8} n_{1,m}^1 + r_{m,8} n_{2,m}^0 + r_{m,8} n_{3,m}^0}{1-\omega}$$

$$\widehat{w} = \sum_{m=1}^{M} \frac{n_{1,m}^1 \pi_1 + n_{2,m}^1 \pi_2 + n_{3,m}^1 \pi_3}{(n_{1,m}^1 + n_{1,m}^0)\pi_1 + (n_{2,m}^1 + n_{2,m}^0)\pi_2 + (n_{3,m}^1 + n_{3,m}^0)\pi_3}$$

where

$$\pi_1 = r_{m,5} + r_{m,6} + r_{m,7} + r_{m,8}$$

$$\pi_2 = r_{m,3} + r_{m,4} + r_{m,7} + r_{m,8}$$

$$\pi_3 = r_{m,2} + r_{m,4} + r_{m,6} + r_{m,8}$$

To estimate p_{ic}:

$$\frac{\partial \hat{Q}}{\partial (p_{1C=k})} = \frac{\partial Q}{\partial (p_{1C=k})} - t = \sum_{M,C_m=k} \frac{r_{m,5} + r_{m,6} + r_{m,7} + r_{m,8}}{p_{1C=k}} - t = 0$$

$$\frac{\partial \hat{Q}}{\partial (p_{2C=k})} = \frac{\partial Q}{\partial (p_{2C=k})} - t = \sum_{M,C_m=k} \frac{r_{m,3} + r_{m,4} + r_{m,7} + r_{m,8}}{p_{2C=k}} - t = 0$$

$$\frac{\partial \hat{Q}}{\partial (p_{3C=k})} = \frac{\partial Q}{\partial (p_{3C=k})} - t = \sum_{M,C_m=k} \frac{r_{m,2} + r_{m,4} + r_{m,6} + r_{m,8}}{p_{3C=k}} - t = 0$$

$$\frac{\partial \hat{Q}}{\partial t} = \sum_{k=1}^{|C|_i} p_{ic} - 1 = 0$$

E-M algorithm for more than 3 species

The E-M algorithm can be generalized to work on arbitrary number of species. If the number of species is A, then there are $B = 2^A$ possible configurations of regulatory states. For each configuration, we could write down the $R_{m,a}$ based on the sequence and the expression models. With all $R_{m,a}$, we could get

$$r_{m,a} = \frac{R_{m,a}}{\sum_{j=1}^{B} R_{m,j}}$$

Then the expectation of the likelihood is:

$$Q(\theta|\theta^t) = \sum_{m=1}^{M} \sum_{j=1}^{B} r_{m,j} \log(R_{m,j})$$

The maximization can be achieved from the derivatives of $Q(\theta|\theta^t)$, which is straightforward.
